# Acetate Activation in *Methanosaeta thermophila*: Characterization of the Key Enzymes Pyrophosphatase and Acetyl-CoA Synthetase

**DOI:** 10.1155/2012/315153

**Published:** 2012-08-15

**Authors:** Stefanie Berger, Cornelia Welte, Uwe Deppenmeier

**Affiliations:** Institute for Microbiology and Biotechnology, University of Bonn, Meckenheimer Allee 168, 53115 Bonn, Germany

## Abstract

The thermophilic methanogen *Methanosaeta thermophila* uses acetate as sole substrate for methanogenesis. It was proposed that the acetate activation reaction that is needed to feed acetate into the methanogenic pathway requires the hydrolysis of two ATP, whereas the acetate activation reaction in *Methanosarcina sp.* is known to require only one ATP. As these organisms live at the thermodynamic limit that sustains life, the acetate activation reaction in *Mt. thermophila* seems too costly and was thus reevaluated. It was found that of the putative acetate activation enzymes one gene encoding an AMP-forming acetyl-CoA synthetase was highly expressed. The corresponding enzyme was purified and characterized in detail. It catalyzed the ATP-dependent formation of acetyl-CoA, AMP, and pyrophosphate (PP_i_) and was only moderately inhibited by PP_i_. The breakdown of PP_i_ was performed by a soluble pyrophosphatase. This enzyme was also purified and characterized. The pyrophosphatase hydrolyzed the major part of PP_i_ (*K*_*M*_ = 0.27 ± 0.05 mM) that was produced in the acetate activation reaction. Activity was not inhibited by nucleotides or PP_i_. However, it cannot be excluded that other PP_i_-dependent enzymes take advantage of the remaining PP_i_ and contribute to the energy balance of the cell.

## 1. Introduction

Methanogenic archaea are of high ecological importance as they are responsible for closure of the global carbon cycle and production of the greenhouse gases CO_2_ and methane [1–3]. They are also an integral part of biogas reactors and contribute to the production of the combustible gas methane that is a source of renewable energy [4, 5]. Methanogenic archaea use end products of anaerobic bacterial degradation processes like H_2_/CO_2_ and acetate as substrates for growth. It is estimated that about two thirds of the methane produced by methanogenic archaea on earth derives from acetate degradation [6]. But despite its high abundance only two genera are able to use acetate as substrate for methanogenesis, namely, *Methanosarcina *and *Methanosaeta*. While *Methanosarcina* species are metabolically versatile, members of the genus *Methanosaeta* are specialized on acetate utilization. This is reflected in a very high affinity for the substrate. For growth, a minimal concentration of only 7–70 *μ*M is needed [7]. Therefore, *Methanosaeta* species prevail over members of the genus *Methanosarcina* in low acetate environments frequently encountered in natural habitats. Important biotechnological habitats are biogas facilities [8–12], where *Methanosaeta* species are of special importance for reactor performance and stability [12, 13].

In acetate-degrading (aceticlastic) methanogenesis, acetate first has to be activated at the expense of ATP. This reaction can be catalysed by the high activity but low affinity acetate kinase/phosphotransacetylase (AK/PTA) system that is used by *Methanosarcina* sp. [14, 15] or by the low-activity but high-affinity AMP-dependent acetyl-CoA-synthetases (ACS) [16–18]. While the AK/PTA system generates ADP, P_i_ and acetyl-CoA from ATP, CoA, and acetate [15, 19, 20], the ACS converts ATP, CoA, and acetate to acetyl-CoA, AMP and pyrophosphate (PP_i_) [16, 18]. In the first step of aceticlastic methanogenesis, acetyl-CoA is cleaved into its methyl and carbonyl moiety by the action of a CO dehydrogenase/acetyl-CoA synthase. In the course of this reaction, the carbonyl group is oxidized to CO_2_ and electrons are transferred to ferredoxin [21–23]. The methyl group is donated to the methanogenic cofactor tetrahydrosarcinapterin and subsequently transferred to coenzyme M (CoM) by a membrane bound Na^+^ translocating methyltransferase. Reduction of the methyl group to methane with coenzyme B as electron donor leads to the formation of the so-called heterodisulfide (CoM-S-S-CoB). Only recently we demonstrated that *Methanosaeta (Mt.) thermophila* uses the heterodisulfide as terminal electron acceptor in an anaerobic respiratory chain with reduced ferredoxin as the sole electron donor [24]. However, the way this organism conserves energy is not yet fully understood. It can be estimated that the amount of ions translocated over the cytoplasmic membrane in the course of aceticlastic methanogenesis could be sufficient for the phosphorylation of two ADP molecules. Yet AMP-dependent acetyl-CoA synthetase and soluble pyrophosphatase (PPiase) activities could be demonstrated for the closely related *Mt. concilii* [16, 18, 25]. Taking non-energy coupled hydrolysis of pyrophosphate into account, two ATP equivalents are consumed in the course of the acetate activation reaction. According to this model, the obligate aceticlastic methanogen *Mt. thermophila* is not able to conserve energy during methanogenesis. To clarify this contradiction, the acetate activation reaction in *Mt. thermophila* was reevaluated by gene expression analysis and characterization of ACS and PPiase.

## 2. Materials and Methods

### 2.1. Materials

All chemicals and reagents were purchased from Sigma-Aldrich (Munich, Germany) or Carl Roth GmbH (Karlsruhe, Germany). Restriction endonucleases, T4 DNA ligase, Taq DNA polymerase, and PCR reagents were purchased from Fermentas (St. Leon-Rot, Germany). Phusion DNA polymerase was purchased from New England Biolabs (Frankfurt am Main, Germany). Oligonucleotides were synthesized by Eurofins (Ebersberg, Germany).

### 2.2. Bioinformatics

For Blast analyses, the respective tool on NCBI (http://www.ncbi.nlm.nih.gov/) was used. For the batch Blast analysis, those proteins that had a threshold *E*-value < e^−40^ were referred to as homologous. The programs PsiPred (http://bioinf.cs.ucl.ac.uk/psipred/) and InterPro (http://www.ebi.ac.uk/interpro/) were utilized for bioinformatic analyses of CBS domains.

### 2.3. qRT-PCR

Total RNA from *Mt. thermophila* DSM 6194 was isolated by TRI Reagent extraction. 250 mL cultures were grown anaerobically to the mid- to late- exponential growth phase in DSMZ medium 387 at 55°C with 50 mM sodium acetate. The cultures were filled into centrifuge tubes in an anaerobic hood and were quick-chilled by shaking in an ice-cold ethanol bath (−70°C) for 5 min. Afterwards, cells were harvested under anaerobic conditions by centrifugation (11000 ×g, 25 min, 4°C). Cell pellets were resuspended in 5 mL TRI Reagent and lysed via a freeze-thaw treatment at −70°C overnight. Total RNA was extracted according to the manufacturer's instructions (Ambion, Darmstadt, Germany). Preparations were treated with DNAse I to reduce DNA contaminations. Cleaning and concentration of RNA were achieved using the SurePrep RNA Cleanup and Concentration kit (Fisher Scientific, Schwerte, Germany). RNA purity was quantified spectrophotometrically by examining the 260 nm/280 nm ratio as well as by denaturing agarose gel electrophoresis.

Primers for qRT-PCR were designed using the Primer3 software (http://frodo.wi.mit.edu/primer3/input.htm). For the highly homologous ACS genes, the least homologous areas were used as templates to guarantee specificity of the primers. The genes encoding glyceraldehyde-3-phosphate dehydrogenase (GAP-DH, *mthe_0701*) and ribosomal protein S3P (*mthe_1722*) were chosen as reference genes. Sequences of the primers used can be seen from Table 1.

PCR reactions were performed according to the manufacturer's instructions (http://www1.qiagen.com/) with on average 250 ng of RNA. The QuantiTect SYBR Green RT-PCR kit (Qiagen, Hilden, Germany) and the iCycler (Bio-Rad, Munich, Germany) were used for labeling and quantification, respectively. For data analysis, the Bio-Rad iCycler software was used. Each PCR product gave a single narrow peak in the melting curve analysis. A relative value (Δ*C*_*t*_) for the initial target concentration in each reaction was determined by subtracting *C*_*t*_ values of the reference genes from those of the genes of interest. By subtracting Δ*C*_*t*_ values, comparisons among the genes of interest could be accomplished. In addition, negative-control assays were included that were not incubated with reverse transcriptase. These assays contained only traces of DNA that were not removed by DNase treatment. The *C*_*t*_ values of the negative controls were analyzed and were at least five cycles higher than the assays with reverse transcriptase treatment.

### 2.4. Cloning into Expression Vectors

Genes from *Mt. thermophila* were amplified from chromosomal DNA extracted with CTAB [26]. Restriction endonuclease sites were inserted by PCR; Primers had the following sequences (recognition sites for restriction endonucleases are underlined): *mthe_0236* for ATGGTAGGTCTCAAATGGCAGATAATATCTATGTGGTCGGG, *mthe_0236* rev ATGGTAGGTCTCAGCGCTCTTCTTGAATGCGGACTCGAGC, *mthe_1194* for ATGGTAACCTGCATTAGCGCCGCTGAGACTGCAAAGACTGCTG, *mthe_1194* rev ATGGTAACCTGCATTATATCAGACTATGAGCGGGATGTTCTCG. For cloning of the pyrophosphatase gene (*mthe_0236*), *Eco*31I was used, for cloning of the AMP-dependent ACS gene (*mthe_1194*) *Bve*I. Amplicons were cut and ligated into pASK-IBA3 or pASK-IBA5 (IBA GmbH, Göttingen, Germany) to produce pASK-*mthe0236*-3 and pASK-*mthe1194*-5, respectively. Both vectors contained plasmid encoded ribosomal binding sites and a Strep-tag II either C-terminal (pASK-IBA3) or N-terminal (pASK-IBA5). The constructs were confirmed by sequencing and transformed into *E. coli *[27].

### 2.5. Protein Overproduction and Purification

Overproduction of proteins was performed in *E. coli* BL21 (DE3) including the plasmid pLysS (Novagen/Merck, Darmstadt, Germany). Cells were grown on modified maximal induction medium [28] with 3.2% [w/v] tryptone, 2% [w/v] yeast extract, and additions of M9 salts as well as 0.1 mM CaCl_2_, 1 mM MgSO_4_ and 1 *μ*M ammonium iron(III) citrate. Ampicillin (100 *μ*g mL^−1^) and chloramphenicol (25 *μ*g mL^−1^) were added for plasmid maintenance. Cultures were grown aerobically at 37°C to an OD_600_ of 0.6; protein production was induced by addition of anhydrotetracyclin (200 ng mL^−1^). Cells were allowed to grow for another 3-4 hours, harvested by centrifugation (11000 ×g, 10 min) and lysed by sonication. Protein purification by Strep-tactin affinity chromatography was performed aerobically according to the manufacturer's instructions (IBA GmbH, Göttingen, Germany). The purified protein was stored at –70°C.

### 2.6. Protein Visualisation

SDS-PAGE was done according to Laemmli [29] with a 5% [w/v] polyacrylamide stacking gel and a 12.5% [w/v] slab gel. Samples were diluted in sample loading buffer (2% [w/v] SDS, 5% [v/v] *β*-mercaptoethanol, 50% [v/v] glycerol, 20% [v/v] collecting buffer (0.625 M Tris-HCl pH 6.8), 0.001% [w/v] bromophenol blue), boiled for 5 min at 95°C and loaded to the gel. Molecular masses were calculated by comparison to a molecular mass standard (Fermentas, St. Leon-Rot, Germany). Proteins were visualized by silver staining [30].

### 2.7. Gel Filtration Chromatography

For gel filtration chromatography a Hi Load 16/60 Superdex 75 prep grade column (GE Healthcare, Munich, Germany) was employed. Calibration was done using the kit for molecular weights, 29000–700000 for gel filtration chromatography (Sigma–Aldrich, Munich, Germany) according to the manufacturer's instructions. For determination of the void volume Blue Dextran was employed. The *K*_av_ was calculated according to

(1) $$\begin{array}{c} K_{\text{av}}\;=\frac{\left(v_{e}-v_{o}\right)}{\left(v_{c}-v_{o}\right)}, \end{array}$$

*v*_*e*_ being the elution volume, *v*_*o*_ the void volume and *v*_*c*_ the column volumn. *K*_av_ was plotted against the decadal logarithm of the molecular weight of the proteins used for calibration, and the resulting curve was used for molecular mass determination. Averaged 1.5 mg of the soluble pyrophosphatase were loaded and run in 40 mM Tris-HCl pH 8, 150 mM NaCl, and 1 mM MnCl_2_ at a rate of 0.5 mL min^−1^.

### 2.8. Enzyme Assays

Assay mixtures for Mthe_0236 routinely contained 200 *μ*L total volume with 40 mM Tris-HCl pH 8, 5 mM MgCl_2_ and 1 mM PP_i_. For measuring the manganese containing enzyme, the protein preparation was pre-incubated for 5 min at room temperature in 40 mM Tris-HCl pH 8, 5 mM MgCl_2_ and 1 mM MnCl_2_ prior to the measurement. For measuring inhibitory effects of nucleotides, either 750 *μ*M AMP or 5 *μ*M ADP were included. For measuring the effect of phosphate between 0 and 1.5 mM, KH_2_PO_4_ were added. The activity of the pyrophosphatase was determined with a discontinuous assay so samples were taken at different time points and the content of the reaction product orthophosphate was measured (modified after Saheki et al. [31]). Values were compared to standard curves (0–2 mM *P*_*i*_). To run more reactions in parallel, tests were performed in 96-well plates. Therefore, 10 *μ*L of sample from the assay mixture were stopped with 2 *μ*L 10% [w/v] trichloroacetic acid. 150 *μ*L of molybdate reagent (15 mM (NH_4_)_6_Mo_7_O_24_, 70 mM zinc acetate, pH 5.0 with HCl) were added as well as 50 *μ*L of 10% [w/v] ascorbic acid (pH 5.0 with NaOH). After incubation at 30°C for 15 min, absorption at 850 nm was measured with the Nanoquant Infinite M200 (Tecan, Männedorf, Switzerland). One unit was defined as *μ*mol PP_i_ hydrolyzed min^−1^.

For measuring the activity of the AMP-dependent ACS (Mthe_1194) two different methods were employed. Temperature stability, the K_M_ value for acetate, and inhibition by PP_i_ were measured via auxiliary enzymes according to a method modified after Meng et al. [32] (Table 2). In this method production of AMP by Mthe_1194 was coupled to NADH consumption that was followed photometrically at 340 nm. In a standard 1 mL assay 50 mM HEPES pH 7.5, 5 mM MgCl_2_, 3 mM phosphoenolpyruvate, 1 mM CoA, 2.5 mM ATP, 1 mM DTT, 20 mM sodium acetate, and 0.15 mM NADH were included. Reaction temperature was set to 55°C. Auxiliary enzymes were sufficiently stable at this temperature, and the amounts of auxiliary enzymes (5.7 U myokinase, 2.3 U pyruvate kinase, 2.1 U lactate dehydrogenase) were not rate limiting. The extinction coefficient of NADH was 6.22 mM^−1 ^cm^−1^. One unit was defined as one *μ*mol of acetate consumed per min that was equal to two *μ*mols of NADH consumed per min.

The K_M_ values for ATP and CoA (reaction volume 3.5 mL) as well as substrate specificity (reaction volume 2 mL) were determined by using a discontinuous assay. At different time points 380 *μ*L samples were taken and the content of PP_i_ was measured according to a method modified after Kuang et al. [33]. The reaction in the samples was stopped with 380 *μ*L 12% TCA [w/v] and 100 *μ*L molybdate reagent (2.5% [w/v] (NH_4_)_6_Mo_7_O_24_ in 5 N H_2_SO_4_), 100 *μ*L 0.5 M *β*-mercaptoethanol and 40 *μ*L Eikonogen reagent were added for detection of PP_i_. The Eikonogen reagent was prepared by dissolving 0.25 g Na_2_SO_3_, 14.65 g KHSO_3_ and 0.25 g 1-amino-2-naphthol-4-sulfonic acid in 100 mL hot water. The solution was cooled down and filtered before use. The reaction mixture for PP_i_ analysis was incubated for 15 min at 37°C and the absorption at 580 nm measured. Quantification was done using standard curves (0–0.5 mM PP_i_). One unit was defined as *μ*mol acetate depleted per min.

## 3. Results

### 3.1. Comparison of Genomes of *Methanosaeta thermophila* and *Methanosarcina mazei*

The recently completed genome sequence of *Mt. thermophila* [23] indicated that the majority of the core steps of aceticlastic methanogenesis are similar in comparison to the genus *Methanosarcina*, but striking differences have been discovered in electron transfer reactions and energy conservation apparatus. These findings led us to a detailed and comprehensive comparison of proteins. A batch Blast analysis of all amino acid sequences from *Ms. mazei* against *Mt. thermophila* and vice versa was performed. In summary, there were about 900 proteins identified that were present in *Ms. mazei* and *Mt. thermophila* (not shown). Among the homologs are enzymes that participate in the central part of aceticlastic methanogenesis and proteins involved in DNA replication, transcription, and translation. Taking into account that the genome of *Mt. thermophila* codes for 1698 proteins, about 800 proteins found in *Mt. thermophila* had no counterpart in *Ms. mazei*. On the other hand, *Ms. mazei* contains 3371 genes indicating that about 2500 proteins can be produced in *Ms. mazei* that are not found in *Mt. thermophila*.

A detailed inspection of the genome of *Mt. thermophila *revealed that the respiratory chain is simpler in comparison to *Methanosarcina* species and is composed only of the F_420_H_2_ dehydrogenase and the heterodisulfide reductase. There are no genes on the chromosome that encode hydrogenases (neither F_420_-reducing hydrogenase (Frh) and F_420_-nonreducing hydrogenase (Vho), nor Ech hydrogenase) [23] or the Rnf complex (encoding a membrane-bound enzyme able to oxidize reduced ferredoxin) [23]. Also membrane fractions of *Mt. thermophila* were shown not to exhibit any hydrogenase activity [24]. In addition, there is no membrane-bound pyrophosphatase and the electron input module of the F_420_H_2_ dehydrogenase FpoF [34] is also missing. Furthermore, genes for acetate kinase and phosphotransacetylase are absent. In contrast to this limited equipment, *Mt. thermophila* possesses four genes encoding acetyl-CoA synthetases (ACS) [23]. No homologs to these four genes are found in *Methanosarcina* species. There was no evidence for a membrane-bound pyrophosphatase that could couple the hydrolysis of pyrophosphate to ion extrusion [35] and thus contribute to energy conservation. Instead, a single soluble type II pyrophosphatase was identified (*mthe_0236*) [23].

### 3.2. Characterization of the Pyrophosphatase

The current hypothesis of the acetate activating reaction in *Methanosaeta* species is that pyrophosphate, produced in the course of acetyl-CoA formation, is hydrolyzed by a pyrophosphatase [25]. However, from our knowledge of the energy conserving system of these organisms it is evident that at least part the energy from the pyrophosphate bond has to be conserved. Therefore, the soluble pyrophosphatase from *Mt. thermophila* was characterized with respect to gene expression and enzyme activity.

The transcript level of the gene encoding the soluble type II pyrophosphatase was analyzed by qRT-PCR experiments. The number of transcripts was three- to four fold higher than that of the reference genes encoding GAP-DH and ribosomal protein S3P (Figure S1). Since at least the gene encoding the S3P protein has to expressed in high amounts for efficient ribosome formation, it is evident that the soluble pyrophosphatase mRNA exists in great copy numbers in cells of *Mt. thermophila*.

Furthermore, the soluble type II pyrophosphatase was found to contain a single CBS domain situated near the N-terminus that could have regulatory effects triggered by binding of ligands such as AMP and ADP [36–38]. Consequently, the pyrophosphatase from *Mt. thermophila* could be potentially inhibited under low-energy conditions (low ATP/ADP ratio) enabling the cell to take advantage of the phosphate group transfer potential of pyrophosphate.

**Figure 1:**
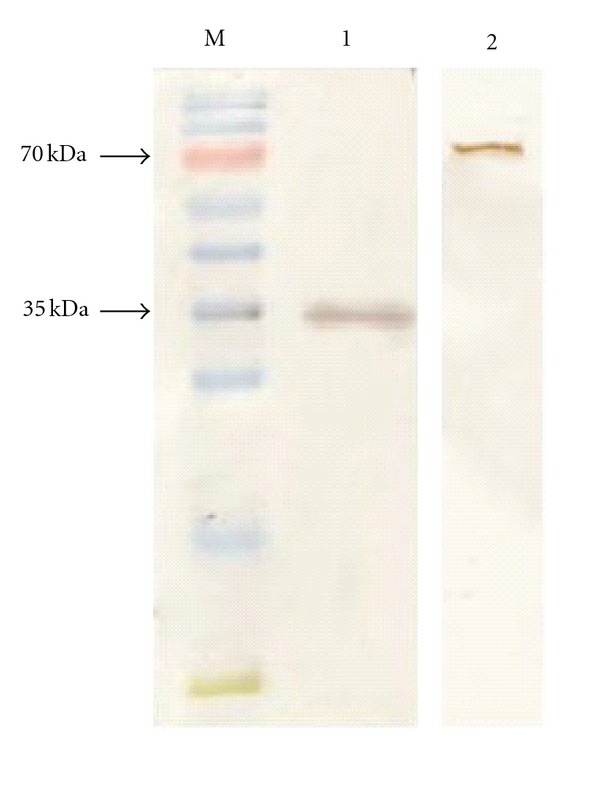
SDS-PAGE analysis of purified soluble pyrophosphatase (Mthe_0236) and AMP-dependent ACS (Mthe_1194). Enzymes were purified by Strep-tactin affinity chromatography. M: molecular mass marker (PAGE Ruler prestained protein ladder, Fermentas, St. Leon-Rot, Germany), lane 1: Mthe_0236 0.5 *μg*, lane 2: Mthe_1194 1 *μ*g.

Blast analyses revealed that CBS domains are rarely found in pyrophosphatases of methanogenic archaea. They could only be identified in soluble pyrophosphatases from species of *Methanosaeta*, *Methanocaldococcus*, and *Methanotorris*. However, biochemical data exists only for the pyrophosphatase from *Mt. concilii *[25]. Thus, functionality of CBS domains in pyrophosphatases of methanogenic archaea has not yet been shown. To evaluate the kinetic parameters of the pyrophosphatase from *Mt. thermophila*, the gene *mthe_0236* was cloned into an expression vector and the respective protein was overproduced in *E. coli*. A single band was detected at 35 kDa on the SDS gel after Strep-tactin affinity purification (Figure 1). This was in accordance with the predicted molecular mass of 35 kDa. The native conformation of the pyrophosphatase was assayed by gel filtration chromatography. The molecular mass of the native enzyme was 71.4 ± 5 kDa. Thus, the native pyrophosphatase was a homodimer. Crystal structures of the soluble type II pyrophosphatases from *Bacillus subtilis*, *Streptococcus gordonii,* and *Streptococcus mutans* revealed that these enzymes are also homodimers in their native conformation [39, 40].

**Figure 2:**
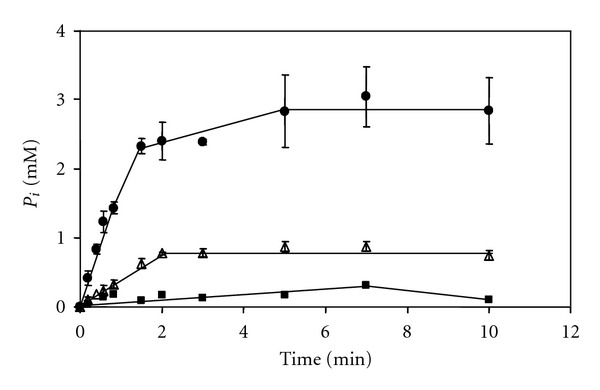
Activity measurement of the soluble type II pyrophosphatase (Mthe_0236). Assays contained 40 mM Tris-HCl pH 8 with 5 mM MgCl_2_ and 1.25 *μ*g enzyme/mL. (●) activity measurement after 5 min preincubation with 1 mM MnCl_2_, (Δ) activity without preincubation, and (■) control without Mthe_0236.

Kinetic analysis showed that the v_max⁡_ of the enzyme was 157 ± 33 U/mg with Mg^2+^ and 726 ± 40 U/mg with Mn^2+^ as metal cofactor (Figure 2). The K_M_-value for PP_i_ was measured with Mn^2+^ as metal ion in the catalytic center and was found to be 0.27 ± 0.05 mM. As indicated earlier, the presence of the CBS domain pair pointed towards a possible regulation of enzyme activity by nucleotides. However, our experiments demonstrated that the pyrophosphatase was not inhibited by nucleotides or its end product phosphate: neither addition of 750 *μ*M AMP or 5 *μ*M ADP nor addition of up to 1.5 mM phosphate led to a reduced reaction rate. Hence, the results indicate that the single CBS domain found in the soluble pyrophosphatase of *Mt. thermophila* is not involved in the regulation of enzyme activity. In contrast, two CBS domains (referred to as Bateman domain [38]) were identified in the membrane-bound pyrophosphatase from the bacterium *Moorella thermoacetica* and inhibition by adenine nucleotides was demonstrated [41].

### 3.3. Characterization of the ACS

The genome of *Mt. thermophila* contains four genes encoding putative AMP-dependent ACS enzymes, three of which are tandemly positioned (*mthe_1194*–*mthe_1196*). The gene encoding the fourth putative ACS is located elsewhere as a single gene (*mthe_1413*). Additionally, we identified a gene encoding a putative ADP-dependent acetyl-CoA-synthetase (*mthe_0554*).

To unravel which of the ACS enzymes catalyzes the acetate activation reaction *in vivo*, the transcript amount of the respective genes was investigated. qRT-PCR experiments demonstrated that* mthe_1194* is the most abundantly expressed of the ACS genes (see Figure S1 in Supplementary Material available online at doi: 10.1155/2012/315153). The transcript level of *mthe_1194* was 2.6 and 2.0 fold higher in comparison to the *gap* gene and the gene encoding the S3P protein, respectively. In contrast, the other ACS encoding genes *mthe_1195* and *mthe_1196 *showed 23-fold and 37-fold reduced transcript concentrations compared to *mthe1194*, respectively. Expression of the single gene *mthe_1413* was only slightly lower than expression of *mthe_1194*, whereas the mRNA content of the putative ADP-dependent acetyl-CoA-synthetase, *mthe_0554*, was about 4000-fold lower under the chosen growth conditions and was near to the detection limit of our assays.

The question arose whether the ACS encoding genes *mthe_1194-1196* are organized in one operon. A closer inspection indicated that the genes are separated by at least 300 bp that contain potential transcriptional starting elements (TATA and BRE boxes). Therefore, the organization of this gene cluster was further analyzed by qRT-PCR using primers pairs that bridged the intergenic regions starting from the beginning and the end of the *acs* genes. With this technique, we could detect mRNA that covered the intergenic regions between the genes. The results of qRT-PCR clearly indicated that the intergenic region between *mthe_1195* and *mthe_1196* was transcribed to the same extent as the genes themselves, indicating that *mthe_1195* and *mthe_1196* were transcribed together (see Figure S1). For the intergenic region between *mthe 1194* and *mthe_1195* no transcript could be detected. Hence, it is highly possible that *mthe_1194* represented a single transcriptional unit.

As the ACS encoded by *mthe_1194* was found to be the most abundantly expressed acetate activation enzyme, it was overexpressed in *E. coli* and the corresponding protein was purified via Strep-tactin affinity chromatography. SDS-PAGE and silver staining revealed a single band at approximately 75 kDa, which was in accordance with the predicted molecular mass (Figure 1).

**Figure 3:**
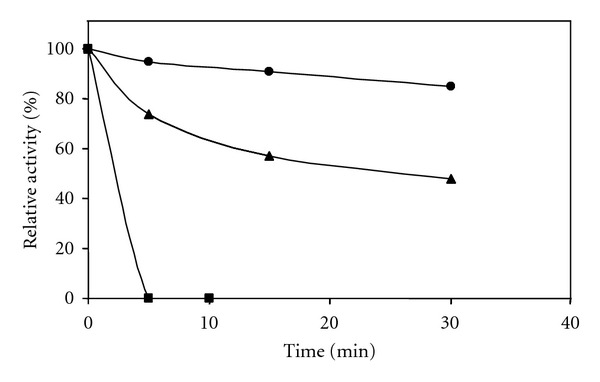
Temperature stability of the AMP-dependent ACS Mthe_1194. (●) Incubation at 55°C, (▲) incubation at 75°C, and (■) incubation at 92°C. Enzyme activity was measured in the NADH consumption assay with auxiliary enzymes at 55°C.

Enzymatic measurements revealed that Mthe_1194 is a thermostable enzyme since 85% of the original activity was retained after incubation at 55°C for 30 min (Figure 3). The optimal growth temperature of *Mt. thermophila* is 55°C and was thus chosen as standard temperature for all enzymatic measurements. In an assay that coupled the formation of AMP to the oxidation of NADH via auxiliary enzymes (see Section 2) a maximal activity of 21.7 U/mg was measured with a *K *_M_ value for acetate at 0.4 mM. An alternate assay utilizing the detection of the pyrophosphate resulted in a maximal activity of 28 U/mg. This test was also used to determine *K *_M_ values for ATP and CoA, which were found to be 20 *μ*M and 14.5 *μ*M, respectively. To differentiate whether the ACS Mthe_1194 was involved only in the activation of acetate in energy metabolism or also in the metabolism of fatty acids, the substrate spectrum was tested. As expected from an enzyme involved in energy metabolism, the enzyme specifically converted acetate to the corresponding thioester. A reaction was also observed with propionate but the specific activity was only 1% compared to the reactivity with acetate. Butyrate did not serve as a substrate for the ACS. It was observed that AMP, ADP, ATP or P_i_ did not inhibit Mthe_1194. In contrast, addition of the final product PP_i_ led to inhibition of enzyme activity. Addition of 0.25 mM pyrophosphate resulted in 50% reduction of the reaction rate.

A central question of the acetate activation reaction in *Mt. thermophila* is whether the energy that is released by the hydrolysis of ATP to AMP and pyrophosphate (2) is sufficient to drive the activation reaction or whether it is necessary to additionally hydrolyse the pyrophosphate to two inorganic phosphates (3) [42]:

(2) ATP→AMP+PPi  ΔG0'=−  31 kJ/mol  [42]

(3) PPi→2  Pi  ΔG0'=−  20 kJ/mol  [42]

Therefore, the activation energy of acetyl-CoA formation by Mthe_1194 was determined. For this purpose, the reaction rate between 20 and 92°C was measured and the natural logarithm of the specific activity plotted against the reciprocal value of the absolute temperature. The activation energy was calculated by using (4) and was 30 kJ/mol.

(4) $$\begin{array}{c} E_{a}=-m\cdot R, \end{array}$$

where *R* is the universal gas constant and *m* is the slope (*R* = 8.314 J mol^−1^ K^−1^, *m* = −3535,5 K^−1^).

## 4. Discussion

Methanogenic archaea performing aceticlastic methanogenesis are living at the thermodynamic limit as the free energy change of this reaction is only −36 kJ/mol. The first step of acetate breakdown is acetate activation. It was proposed that this step differs in the two genera that are able to grow on acetate, *Methanosarcina* and *Methanosaeta*. For *Methanosarcina* sp., it is well established that the acetate kinase/phosphotransacetylase system is used for acetate activation [43, 44]. It is of bacterial origin and was acquired by *Methanosarcina* sp. by lateral gene transfer [45]. In this pathway acetate is activated to acetyl phosphate with concomitant hydrolysis of ATP to ADP and phosphate. In the subsequent step, acetyl phosphate is transformed into acetyl-CoA without further expense of ATP. In total, one ATP equivalent is hydrolyzed. For *Methanosaeta* sp., however, the acetate activation reaction is more ambiguous. The genome sequences of *Mt. concilii* and *Mt. thermophila* indicate that the acetate kinase/phosphotransacetylase enzyme system is absent in these organisms [23, 46]. In addition, these enzyme activities could not be found in cell extract of *Mt. concilii *[17]. Instead, it was proposed that an AMP-dependent acetyl-CoA synthetase should catalyze this reaction [18]. Jetten et al. purified one of these enzymes from *Mt. concilii* [18] that converts acetate to acetyl-CoA and thereby hydrolyzes ATP to AMP and PP_i_. Together with the activity of a soluble pyrophosphatase that was purified by the same group [25] this mode of activation requires the hydrolysis of two ATP equivalents. However, the anaerobic respiratory chain of *Methanosaeta sp*. is purported to be incapable of supporting the generation of more than two ATP molecules from one acetate molecule [24]. Hence, it is rather intriguing how these organisms generate metabolic energy for growth. To overcome this contradiction, we reevaluated the acetate activation reaction in *Mt. thermophila*.

In the genome of *Mt. thermophila*, five different putative ACS enzymes are encoded, four are annotated as AMP-dependent and one as ADP-dependent. So far ADP-dependent acetyl-CoA-synthetases have never been shown to work in the direction of acetyl-CoA formation *in vivo*. Nevertheless, this possibility was considered due to its energetic benefit to the cell. However, qRT-PCR experiments clearly demonstrated that the respective gene is not expressed during the exponential growth phase. Therefore, and because the acetate kinase/phosphotransacetylase system is missing, acetate activation in *Mt. thermophila* is probably catalyzed by an AMP-dependent ACS. It could be shown that one of the four genes encoding AMP-dependent ACS, *mthe_1194*, was the most abundantly expressed. Therefore, the corresponding protein was overproduced and characterized. Involvement in energy metabolism was verified by the fact that acetate is by far the best substrate, which could also be demonstrated for the ACS enzyme from *Methanothermobacter thermoautotrophicus* [47]. Also the K_M_ value for ATP was low, which means that acetate activation by Mthe_1194 is possible even under low-energy conditions. Inhibition by AMP has been shown but did not occur in this case [18, 48]. Instead PP_i_ that is the other reaction product and has also been shown to inhibit ACS enzymes [18, 48] could reduce the reaction rate by 50% at a concentration of 0.25 mM. Thus, accumulation of excess acetyl-CoA along with ATP consumption is avoided in the cytoplasm of *Mt. thermophila*.

The close relative *Mt. concilii* contains five genes encoding putative AMP-dependent ACS enzymes [46]. An ACS from *Mt. concilii *was previously purified from cell extracts and characterized and showed similar enzyme properties to the ACS characterized in this study [18]. However, in light of the recent genome sequencing, it is not certain which of the five ACS isozymes was purified from *Mt. concilii*, or if a mixture of the five highly homologous enzymes (≥58% identity) was obtained.

The finding that PP_i_ is generated during the acetate activation reaction led to the question if the energy released during hydrolysis of PP_i_ is dissipated as heat or if it is (at least in part) used for energy conservation. As indicated above, the genome of *Mt. thermophila* contains only one pyrophosphatase gene, coding for a soluble type II pyrophosphatase (Mthe_0236). We heterologously overproduced the enzyme in *E. coli* and the biochemical characterization indicated that the enzyme indeed possessed the characteristics of a soluble type II pyrophosphatase.

The result of gel filtration chromatography showed that the pyrophosphatase from *Mt. thermophila* is active as a homodimer. In contrast, the pyrophosphatase purified from *Mt. concilii* was found to be a heterotetramer by gel filtration chromatography and SDS-PAGE analysis [25]. However, there is only one gene encoding a soluble type II pyrophosphatase in the genome of *Mt. concilii* [46] that appears as a single transcription unit and is not part of an operon structure. It is tempting to speculate that in *Mt. concilii* a posttranslational modification takes place and two forms of the protein are produced or the smaller protein is a result of a proteolytic cleavage as a first stage of degradation in a normal turn-over process. A native conformation with three to four subunits was found for the type II soluble pyrophosphatase from *Methanocaldococcus jannaschii *[49]. In contrast, soluble type II pyrophosphatase purified from bacteria are made of a single subunits and form homodimers [39, 40]. Hence, the pyrophosphatase from *Mt. thermophila* resembles bacterial enzymes with respect to subunit composition.

Pyrophosphate is formed in enzymatic reactions of various metabolic pathways (e.g., DNA, RNA, and protein biosynthesis) and is supposed to be subsequently hydrolyzed by pyrophosphatases to shift the overall reaction equilibrium towards product formation. However, this view may be too restrictive because a considerable amount of metabolic energy is lost and released as heat. Instead, it might be possible that some of the energy of the PP_i_ anhydride bond could be conserved. For example, by coupling the hydrolysis of PP_i_ to the phosphorylation of cellular compounds thereby forming energy-rich intermediates for biosynthesis. Such enzymes have already been detected in many organisms, such as PP_i_-dependent phosphofructokinases from the sulphur-reducing archaeon *Thermoproteus tenax* [50], bacteria like *Methylococcus capsulatus *[51], *Methylomicrobium alcaliphilum* [52], *Borrelia burgdorferi* [53], and the protozoan *Entamoeba histolytica *[54]. Another prominent example is the pyruvate phosphate dikinase, catalyzing the reversible reaction between pyruvate, ATP and phosphate to phosphoenolpyruvate, AMP, and pyrophosphate. Among others it has been found in *T. tenax* [55], *Bacteroides symbiosis* [56], and *Microbispora rosea *[57]. Genes encoding PP_i_-dependent kinases were not yet annotated in the genome of *Mt. thermophila*. However, the deduced amino acid sequence from gene *mthe_1637* revealed a low but significant homology (e-value of 2 × e^−40^) to the pyruvate phosphate dikinase from *T. tenax*.

The question whether PP_i_ is completely hydrolyzed by the pyrophosphatase in *Mt. thermophila* or whether part of the energy-rich molecule is used for phosphorylation reactions is not clear and will be examined in the future. However, the kinetic parameters of the pyrophosphatase from *Mt. thermophila* are intriguing. The K_M_ values for PP_i_ of the above-mentioned pyrophosphate-scavenging enzymes generally range between 0.2 and 0.015 mM and are thus below the *K *_M_ of 0.3 mM of the pyrophosphatase described here. That means that these could take advantage of part of the pyrophosphate that is released during the process of aceticlastic methanogenesis and thus contribute to the generation of high group transfer potential intermediates that would subsequently contribute to energy conservation.

In summary, it could be shown that the acetate activation reaction in *Mt. thermophila* requires two ATP equivalents per molecule of acetate. It cannot be excluded that part of PP_i_ generated in this process might be used by an unknown enzyme to transfer phosphate groups to an intermediary metabolite. Further investigation into the energy conservation mechanisms of *Methanosaeta* sp. is needed to understand how these organisms that live close to the thermodynamic limits of life can thrive.

## Supplementary Material

Figure S1: Transcript abundance of genes encoding acetyl-CoA synthetases (ACS) and pyrophosphatase (PPiase) from *Mt. thermophila*. Grey boxes: transcript ratio of indicated gene versus gene encoding the glyceraldehyde-phosphate dehydrogenase *gap*; white boxes, transcript ratio of indicated gene versus gene encoding ribosomal protein *S3P*. *mthe_0236 = PPiase; mthe_1194 = acs1; mthe_1195 = acs2; mthe_1196 = acs3, mthe_1413 = acs4*

## Figures and Tables

**Table 1:** Gene number, function of corresponding protein and primers used for amplification of genes analyzed by qRT-PCR.

Function	Gene number	Primer sequence
AMP-dependent ACS	mthe_1194	for CCAGTGGATCATCGAGTA
		rev CAGAAATCGAGGTAGTTC
	mthe_1195	for TAAGGAGCTTGCTGAGAA
		rev CAGAACTCTATGTAGTGG
	mthe_1196	for TCGAAGGCGTATGCTGAC
		rev CGCCTCGTCAGCCTGCTT
	mthe_1413	for CAGGCGCGCTCCGCGAG
		rev GGCCTTTATCGGGATAGG

ADP-dependent ACS	mthe_0554	for TATCATTGGGGTTACAAG
		rev CAGAGATGGGTATTGATC

PPiase	mthe_0236	for GCCAGCATGTATGAGCTG
		rev CATGTGGGTGACTTGAAT

GAP-DH	mthe_0701	for CTATGCCGTTGCTGTGAA
		rev TTGGCGGTGCATTTATCT

ribosomal protein S3P	mthe_1722	for GTTCGTCATGATTGGCAC
		rev CCCCTTCTGGAGCTTATC

intergenic region	Between mthe_1194	for GCGGTCAACCTATTTTATTT
	and mthe_1195	rev TTACATACCTCCATTCATCT

intergenic region	Between mthe_1195	for AACGTCCGCAATTTTTATTT
	and mthe_1196	rev CTGCCTCCAGCCCATCCCG

**Table 2:** Activity measurement of the acetyl-CoA synthetase via auxiliary enzymes. The decrease of the absorption of NADH was tracked photometrically at 340 nm.

Enzyme	Reaction catalyzed
Acetyl-CoA synthetase	acetate + ATP + CoA ⇌ acetyl-CoA + PP_i_+ AMP
Myokinase	AMP + ATP ⇌ 2 ADP
Pyruvate kinase	ADP + PEP ⇌ pyruvate + ATP
Lactate dehydrogenase	pyruvate + NADH ⇌ lactate + NAD^+^
