# Supplementary material for: Acetate Activation in Methanosaeta thermophila: Characterization of the Key Enzymes Pyrophosphatase and Acetyl-CoA Synthetase
Source: Archaea. 2012 Aug 15;2012:315153. doi: 10.1155/2012/315153 (PMC3426162; doi:10.1155/2012/315153)
Supplement: Supplementary file 1 — Figure S1: Transcript abundance of genes encoding acetyl-CoA synthetases (ACS) and pyrophosphatase (PPiase) from Mt. thermophila. Grey boxes: transcript ratio of indicated gene versus gene encoding the glyceraldehyde-phosphate dehydrogenase gap; white boxes, transcript ratio of indicated gene versus gene encoding ribosomal protein S3P. mthe_0236 = PPiase; mthe_1194 = acs1; mthe_1195 = acs2; mthe_1196 = acs3, mthe_1413 = acs4 [file 315153.f1.ppt]

## Slide 1
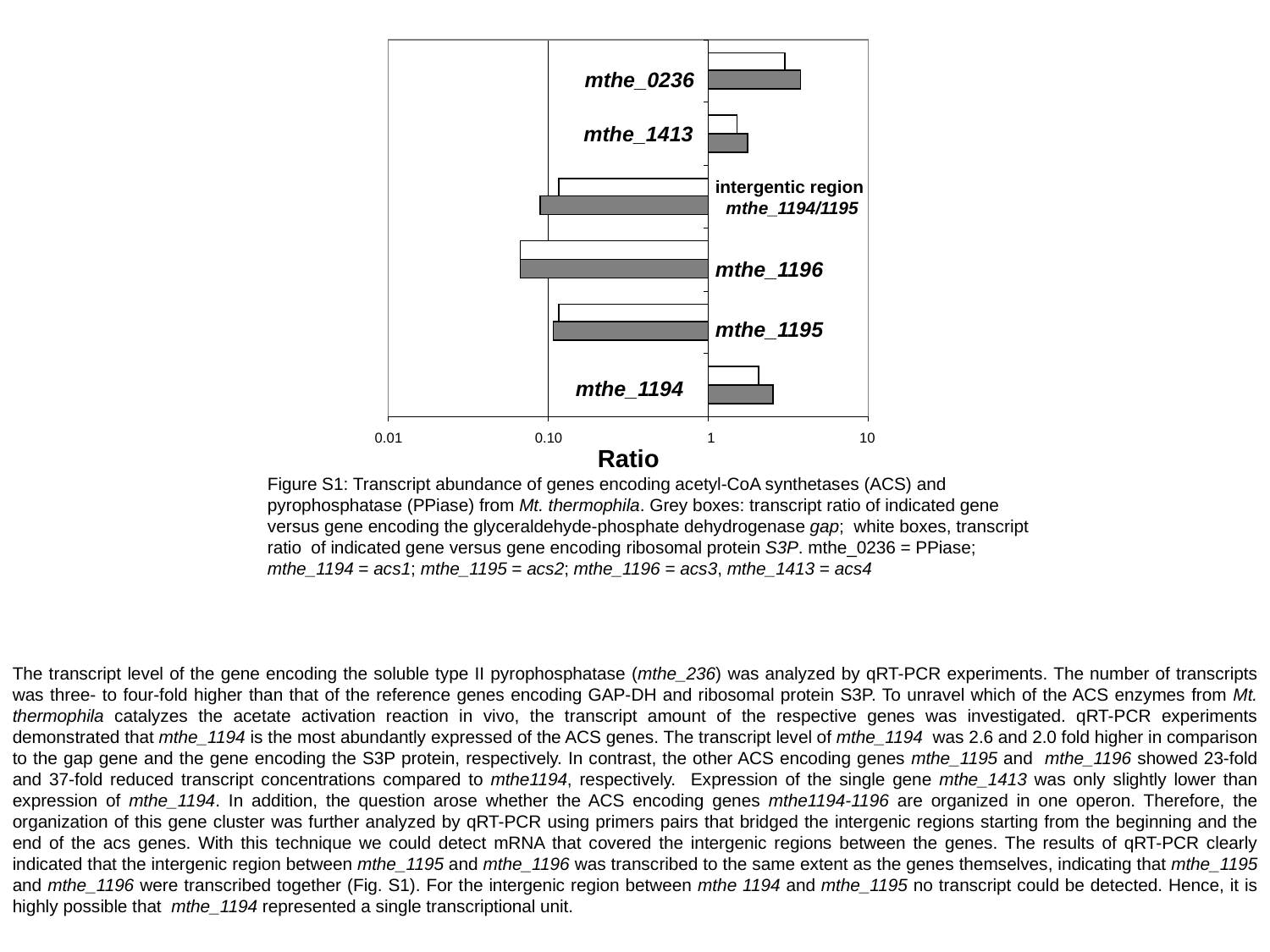

mthe_0236
mthe_1413
intergentic region
mthe_1194/1195
mthe_1196
mthe_1195
mthe_1194
0.01
0.10
1
10
Ratio
Figure S1: Transcript abundance of genes encoding acetyl-CoA synthetases (ACS) and pyrophosphatase (PPiase) from Mt. thermophila. Grey boxes: transcript ratio of indicated gene versus gene encoding the glyceraldehyde-phosphate dehydrogenase gap; white boxes, transcript ratio of indicated gene versus gene encoding ribosomal protein S3P. mthe_0236 = PPiase; mthe_1194 = acs1; mthe_1195 = acs2; mthe_1196 = acs3, mthe_1413 = acs4
The transcript level of the gene encoding the soluble type II pyrophosphatase (mthe_236) was analyzed by qRT-PCR experiments. The number of transcripts was three- to four-fold higher than that of the reference genes encoding GAP-DH and ribosomal protein S3P. To unravel which of the ACS enzymes from Mt. thermophila catalyzes the acetate activation reaction in vivo, the transcript amount of the respective genes was investigated. qRT-PCR experiments demonstrated that mthe_1194 is the most abundantly expressed of the ACS genes. The transcript level of mthe_1194 was 2.6 and 2.0 fold higher in comparison to the gap gene and the gene encoding the S3P protein, respectively. In contrast, the other ACS encoding genes mthe_1195 and mthe_1196 showed 23-fold and 37-fold reduced transcript concentrations compared to mthe1194, respectively. Expression of the single gene mthe_1413 was only slightly lower than expression of mthe_1194. In addition, the question arose whether the ACS encoding genes mthe1194-1196 are organized in one operon. Therefore, the organization of this gene cluster was further analyzed by qRT-PCR using primers pairs that bridged the intergenic regions starting from the beginning and the end of the acs genes. With this technique we could detect mRNA that covered the intergenic regions between the genes. The results of qRT-PCR clearly indicated that the intergenic region between mthe_1195 and mthe_1196 was transcribed to the same extent as the genes themselves, indicating that mthe_1195 and mthe_1196 were transcribed together (Fig. S1). For the intergenic region between mthe 1194 and mthe_1195 no transcript could be detected. Hence, it is highly possible that mthe_1194 represented a single transcriptional unit.
